# Functional predication of differentially expressed circRNAs/lncRNAs in the prefrontal cortex of Nrf2-knockout mice

**DOI:** 10.18632/aging.202688

**Published:** 2021-03-10

**Authors:** Yan-Jing Gao, Run-Jiao Zhang, Qing Liu, Shao-Guang Sun, Mao-Yang Qi, Yue Wang, Dan-Dan Geng, Lei Wang

**Affiliations:** 1 Department of Human Anatomy, Institute of Medicine and Health, Hebei Medical University, Shijiazhuang 050017, Hebei, China; 2 Department of Biochemistry and Molecular Biology, Hebei Medical University, Shijiazhuang 050017, Hebei, China; 3 School of Basic Medicine, Hebei Medical University, Shijiazhuang 050017, Hebei, China

**Keywords:** Nrf2, circular RNA, long non-coding RNA

## Abstract

In the central nervous system, nuclear factor erythroid-2-related factor 2 (Nrf2) protects neurons from oxidant injury, thereby ameliorating neurodegeneration. We explored the key circular RNAs (circRNAs) and long non-coding RNAs (lncRNAs) involved in Nrf2-induced neuroprotection. We used microarrays to examine the circRNAs (DEcircRNAs), lncRNAs (DElncRNAs) and mRNAs (DEmRNAs) differentially expressed between Nrf2 (+/+) and Nrf2 (-/-) mice and identified DEcircRNA/DElncRNA-miRNA-DEmRNA interaction networks. In total, 197 DEcircRNAs, 685 DElncRNAs and 356 DEmRNAs were identified in prefrontal cortical tissues from Nrf2 (-/-) mice. The expression patterns of selected DEcircRNAs (except for mmu_circ_0003404) and DElncRNAs in qRT-PCR analyses were generally consistent with the microarray analysis results. Functional annotation of the DEmRNAs in the DEcircRNA/DElncRNA-miRNA-DEmRNA networks indicated that five non-coding RNAs (mmu_circ_0000233, ENSMUST00000204847, NONMMUT024778, NONMMUT132160 and NONMMUT132168) may contribute to Nrf2 activity, with the help of mmu_circ_0015035 and NONMMUT127961. The results also revealed that four non-coding RNAs (cicRNA.20127, mmu_circ_0012936, ENSMUST00000194077 and NONMMUT109267) may influence glutathione metabolism. Additionally, 44 DEcircRNAs and 7 DElncRNAs were found to possess coding potential. These findings provide clues to the molecular pathways through which Nrf2 protects neurons.

## INTRODUCTION

Nuclear factor erythroid-2-related factor 2 (Nrf2/NFE2L2) is a pleiotropic transcription factor, and studies in mouse models have indicated that Nrf2 influences the susceptibility to diseases ranging from neurodegenerative disorders to endotoxin-induced septic shock [1–3]. Nrf2 belongs to a subset of basic leucine-zipper genes sharing a conserved CNC (cap ‘n’ collar) structural domain [4]. The mammalian CNC family includes four closely related proteins (p45-NFE2, Nrf1, Nrf2 and Nrf3) and two distantly related proteins (Bach1 and Bach2) that function as heterodimeric transcription factors by pairing with other basic leucine-zipper proteins, including the small-Mafs [4]. In the central nervous system, Nrf2 protects against oxidative stress, improves mitochondrial function, inhibits the unfolded protein responses, promotes autophagy, etc [5–10].

Non-coding RNAs (ncRNAs), including circular RNAs (circRNAs), long non-coding RNAs (lncRNAs) and microRNAs (miRNAs), are the dominant products of eukaryotic transcription, and function directly at the RNA level [11–13]. NcRNAs not only are involved in cell proliferation, apoptosis, differentiation, metabolism and other physiological processes, but also contribute to the pathogenesis of diseases [14–18]. LncRNAs are endogenous linear ncRNAs greater than 200 nucleotides in length. CircRNAs lack 5' caps and 3' tails, and thus are resistant to degradation by RNA exonuclease and are much more stable than linear isoforms [19]. CircRNAs and lncRNAs may share certain mechanisms of target gene regulation, including binding to miRNAs as competitive endogenous RNAs (ceRNAs) [20, 21], altering gene transcription, binding to proteins and encoding peptides/proteins [22].

In previous studies, we examined the effects of Nrf2-related circRNAs and lncRNAs on the brain by detecting the altered expression of circRNAs, lncRNAs and mRNAs in different brain areas in Nrf2-null (Nrf2 (-/-)) mice, including substantia nigra, corpus striatum and hippocampus [23–25]. The prefrontal cortex has often been called the command and control center of the brain, and is mainly responsible for cognitive control, especially the ability to orchestrate thought and action in accordance with internal goals [26]. Thus, in this study, we selected the prefrontal cortex to continue this series of studies in Nrf2 (-/-) mice. First, we performed microarray analyses to identify differentially expressed circRNAs (DEcircRNAs), lncRNAs (DElncRNAs) and mRNAs (DEmRNAs) between prefrontal cortex tissues from Nrf2 (-/-) and Nrf2 (+/+) mice. Then, we constructed DEcircRNA/DElncRNA-miRNA-DEmRNA networks and performed Gene Ontology (GO) and Kyoto Encyclopedia of Genes and Genomes (KEGG) pathway analyses to annotate the functions of the DEmRNAs in the networks. Additionally, we evaluated the coding potential of the DEcircRNAs/DElncRNAs, the expression correlations between the DEcircRNAs/DElncRNAs and their host mRNAs, and the intersections between the DEcircRNAs/DElncRNAs/DEmRNAs in different brain areas. Our study has provided clues into the molecular pathways whereby Nrf2 protects neurons.

## RESULTS

### DEcircRNAs, DElncRNAs and DEmRNAs between the prefrontal cortexes of Nrf2 (-/-) and Nrf2 (+/+) mice

**Figure 1:**
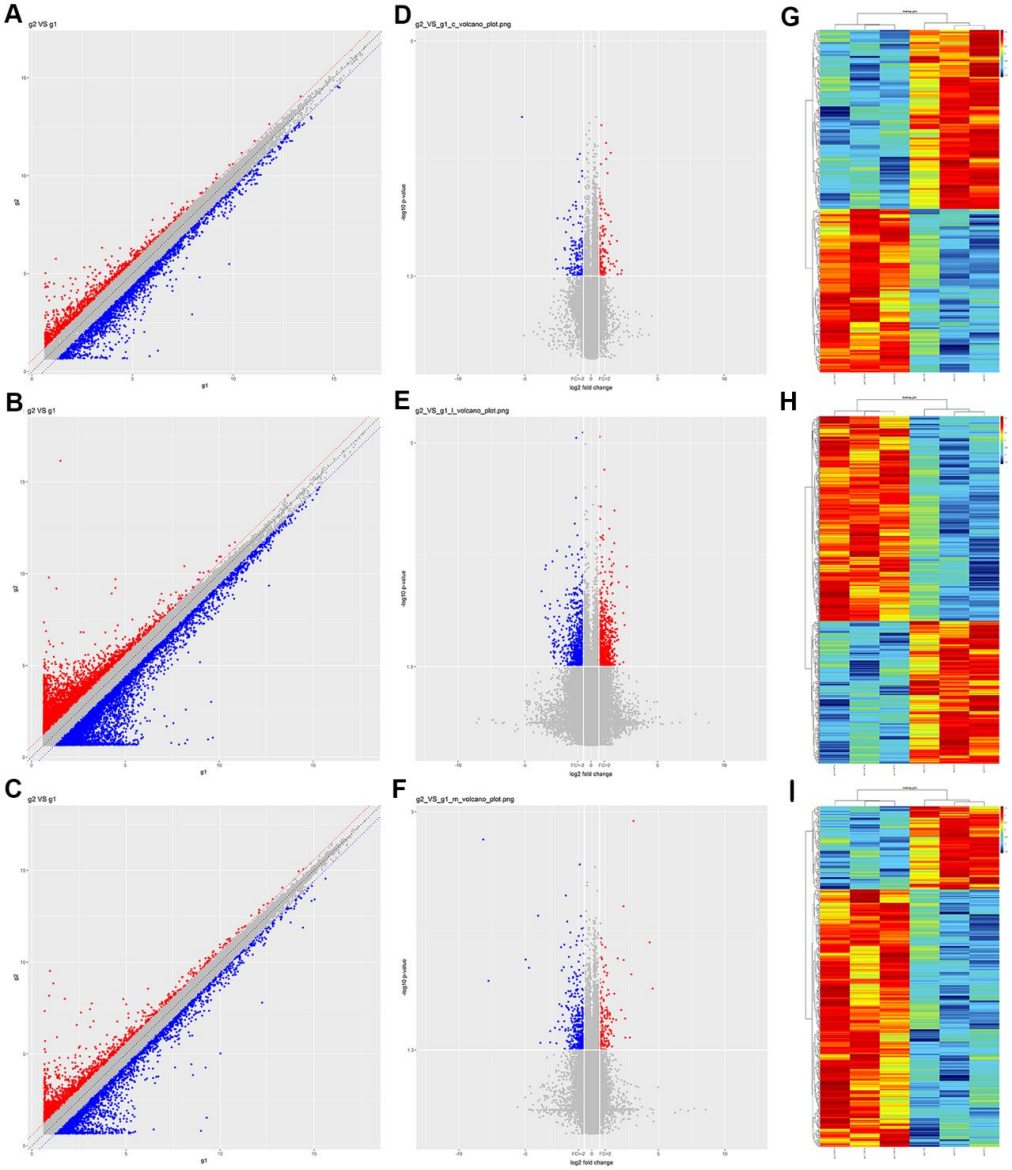
**Expression profiles of circRNAs, lncRNAs and mRNAs in prefrontal cortex tissues from Nrf2 (-/-) and Nrf2 (+/+) mice.** (**A**–**C**) Scatter plots showing the expression variation of circRNAs (**A**), lncRNAs (**B**) and mRNAs (**C**) between Nrf2 (-/-) and Nrf2 (+/+) prefrontal cortex tissues. The values of the X and Y axes in the scatter plots are the normalized signal values of the samples (log_2_-scaled). The red (up-regulated) and blue (down-regulated) points represent the DEcircRNAs (**A**), DElncRNAs (**B**) and DEmRNAs (**C**) with more than FCs > 1.5 between Nrf2 (-/-) and Nrf2 (+/+) prefrontal cortex tissues. (**D**–**F**) Volcano plots showing the differential expression of circRNAs (**D**), lncRNAs (**E**) and mRNAs (**F**) between Nrf2 (-/-) and Nrf2 (+/+) prefrontal cortex tissues. The vertical lines correspond to 1.5-fold upregulation and downregulation, and the horizontal line represents a *p*-value of 0.05. The red (up-regulated) and blue (down-regulated) points represent the DEcircRNAs (**D**), DElncRNAs (**E**) and DEmRNAs (**F**) with statistical significance. g1: group 1, refers to the Nrf2 (+/+) group. g2: group 2, refers to the Nrf2 (-/-) group. (**G**–**I**) Hierarchical clustering analyses were performed to depict the DEcircRANs (**G**), DElncRNAs (**H**) and DEmRNAs (**I**) in Nrf2 (-/-) prefrontal cortex compared with Nrf2 (+/+) prefrontal cortex tissues. The clustering analysis was used to group samples based on their expression values so that the relationships among samples could be predicted. ‘Red’ denotes high relative expression, and ‘green’ denotes low relative expression.

After identifying the genotypes of the mice (Supplementary Figure 1), we performed microarray analyses to detect DEcircRNAs, DElncRNAs and DEmRNAs between the prefrontal cortex of Nrf2 (-/-) and Nrf2 (+/+) mice. The raw data have been deposited in the Gene Expression Omnibus database (https://www.ncbi.nlm.nih.gov/geo/query/acc.cgi?acc=GSE162315). In total, 197 DEcircRNAs with *p*-value < 0.05 and fold-changes (FCs) > 1.5 were detected in the prefrontal cortex, of which 103 up-regulated and 94 down-regulated in Nrf2 (-/-) mice compared with Nrf2 (+/+) mice. Among them, cicRNA.17816 and cicRNA.24872 were the most significantly up- and down-regulated genes, respectively (Table 1). Additionally, 685 DElncRNAs (281 up-regulated and 404 down-regulated in Nrf2 (-/-) mice) were identified, of which NONMMUT132168 and ENSMUST00000195276 were the most significantly up- and down-regulated genes, respectively (Table 2). Moreover, 356 DEmRNAs were detected, of which 87 up- and 269 down-regulated in Nrf2 (-/-) mice. Scatter plots (Figure 1A–1C), volcano diagrams (Figure 1D–1F) and hierarchical clustering analyses (Figure 1G–1I) were used to depict the DEcircRNAs, DElncRNAs and DEmRNAs between the prefrontal cortex of Nrf2 (-/-) and Nrf2 (+/+) mice.

**Table 1:** Top 10 up- and down-regulated DEcircRNAs in the prefrontal cortex of Nrf2 (-/-) mice.

**circRNA**	**chrom**	**Foldchange**	**Regulation**
cicRNA.17816	chr1	2.900872	up
cicRNA.2059	chr17	2.751136	up
cicRNA.22457	chr4	2.491297	up
cicRNA.25732	chr2	2.194124	up
cicRNA.27857	chr19	2.177916	up
cicRNA.10101	chr12	2.130815	up
mmu_circ_0000233	chr11	2.049909	up
cicRNA.14293	chr10	2.034235	up
cicRNA.12176	chr11	2.023146	up
cicRNA.24048	chr3	2.015869	up
cicRNA.24872	chr2	36.804170	down
cicRNA.14175	chr10	5.765533	down
cicRNA.21621	chr5	4.358284	down
cicRNA.20127	chr8	4.081641	down
mmu_circ_0002377	chr10	3.477857	down
cicRNA.16195	chr1	3.138005	down
cicRNA.9167	chr13	2.993984	down
cicRNA.2029	chr17	2.849510	down
cicRNA.13394	chr11	2.782886	down
cicRNA.6318	chr15	2.727061	down

DEcircRNAs, differentially expressed circRNAs.

**Table 2:** Top 10 up- and down-regulated DElncRNAs in the prefrontal cortex of Nrf2 (-/-) mice.

**lncRNA**	**chrom**	**Foldchange**	**Regulation**
NONMMUT132168	chr9	6.486807	up
NR_038039	chr10	6.417579	up
NONMMUT127961	chr7	5.724120	up
NONMMUT132160	chr9	5.327114	up
NONMMUT077500	chr1	4.428169	up
NR_045161	chr13	3.619127	up
NONMMUT122421	chr6	3.489428	up
NONMMUT132149	chr9	3.395637	up
NONMMUT125225	chr7	3.084584	up
NONMMUT083894	chr11	3.048123	up
ENSMUST00000195276	chr4	13.210466	down
NONMMUT118467	chr5	11.100098	down
NONMMUT114173	chr3	10.866240	down
NONMMUT132224	chr9	10.746878	down
ENSMUST00000194077	chr3	10.500292	down
NONMMUT105493	chr19	9.502475	down
NONMMUT000100	chr1	8.942628	down
NONMMUT124473	chr6	8.569627	down
NONMMUT106900	chr2	8.519742	down
NONMMUT076758	chr1	8.509335	down

DElncRNAs, differentially expressed lncRNAs.

### Quantitative real-time polymerase chain reaction (qRT-PCR) validation of DEcircRNA and DElncRNA expression

Considering that DEcircRNAs and DElncRNAs with higher FCs and lower *p*-values were more likely to be regulated by Nrf2, and that higher expression may enable more accurate microarray detection, we selected DEcircRNAs and DElncRNAs with relatively high FCs, high expression values and low *p*-value to validate the accuracy of the microarray data using qRT-PCR. We used different screening criteria to screen different RNAs. For DElncRNAs, those with *p*-values < 0.015, FCs > 2.8 or < 0.15, and Standardized Expression Levels (SELs) > 1 were selected. For DEcircRNAs included in “circBase”, those with *p*-values < 0.05, FCs >1.7 or < 0.55, and SELs > 1 were selected. For DEcircRNAs not included in “circBase”, those with *p*-values < 0.01, FCs >2.5 or < 0.25, and SELs > 1 were selected.

**Figure 2:**
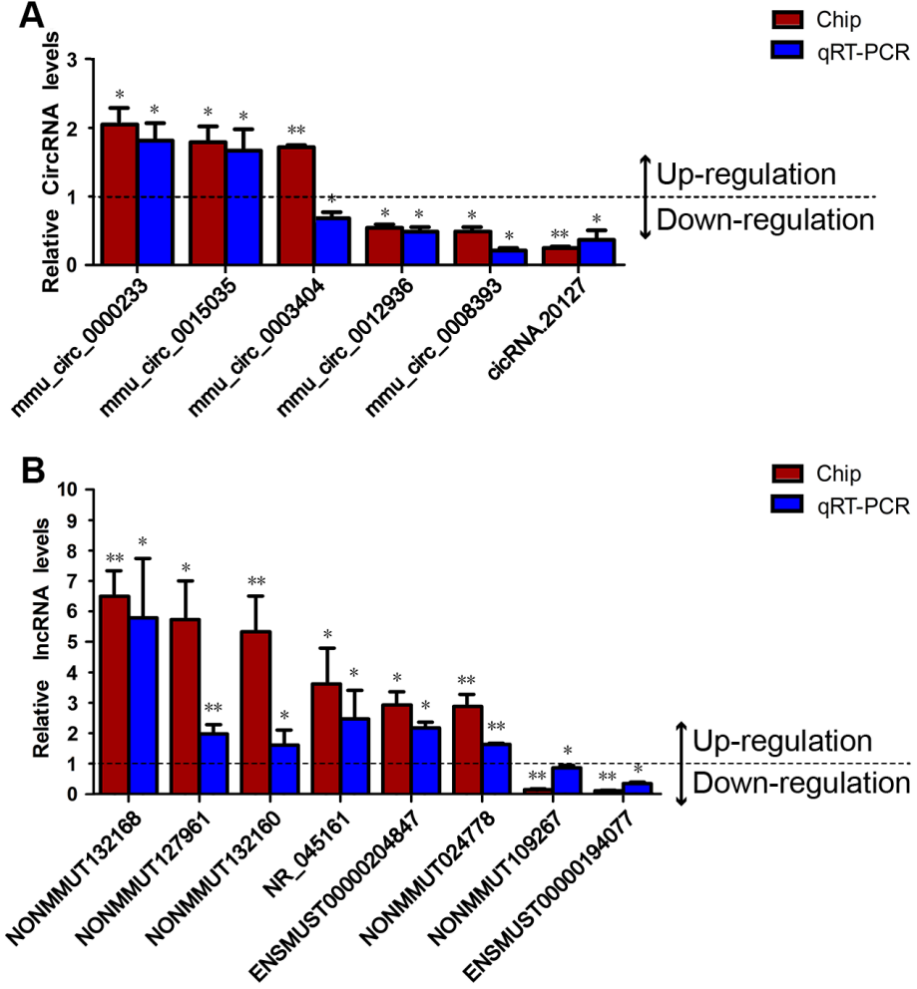
**Validation of the DEcircRNAs and DElncRNAs.** The levels of six DEcircRNAs and eight DElncRNAs were validated in prefrontal cortex samples from Nrf2 (-/-) and Nrf2 (+/+) mice using qRT-PCR and normalized to the internal reference gene (β-actin). (**A**) The qRT-PCR results for five of the verified circRNAs (all but mmu_circ_0003404) were consistent with the microarray results. (**B**) The qRT-PCR results for all eight verified lncRNAs were consistent with the microarray results. All experiments were replicated three times. The deep red column indicates the FCs of the DEcircRNAs/DElncRNAs determined through microarray analyses; the blue column indicates the expression FCs of the DEcircRNAs/DElncRNAs determined through qRT-PCR experiments. The presented values are the means ± standard deviations. Values > 1 indicate up-regulated DEcircRNAs/DElncRNAs, and values < 1 indicate down-regulated DEcircRNAs/DElncRNAs. *p < 0.05, **p < 0.01.

Subsequently, qRT-PCR was used to screen primers, and only six DEcircRNAs and eight DElncRNAs were found to have suitable primers. Thus, three up-regulated DEcircRNAs (mmu_circ_0000233, mmu_circ_0015035 and mmu_circ_0003404), three down-regulated DEcircRNAs (mmu_circ_0012936, mmu_circ_0008393 and cicRNA.20127), six up-regulated DElncRNAs (NONMMUT132168, NONMMUT127961, NONMMUT132160, NR_045161, ENSMUST00000204847 and NONMMUT024778) and two down-regulated DElncRNAs (NONMMUT109267 and ENSMUST00000194077) with relatively high FCs, high expression values and low *p*-values were selected to validate the accuracy of the microarray data using qRT-PCR. As shown in Figure 2, the qRT-PCR results for all the DElncRNAs and DEcircRNAs except for mmu_circ_0003404 were consistent with the microarray results.

### Construction of the DEcircRNA/DElncRNA-miRNA-DEmRNA crosstalk networks

**Figure 3:**
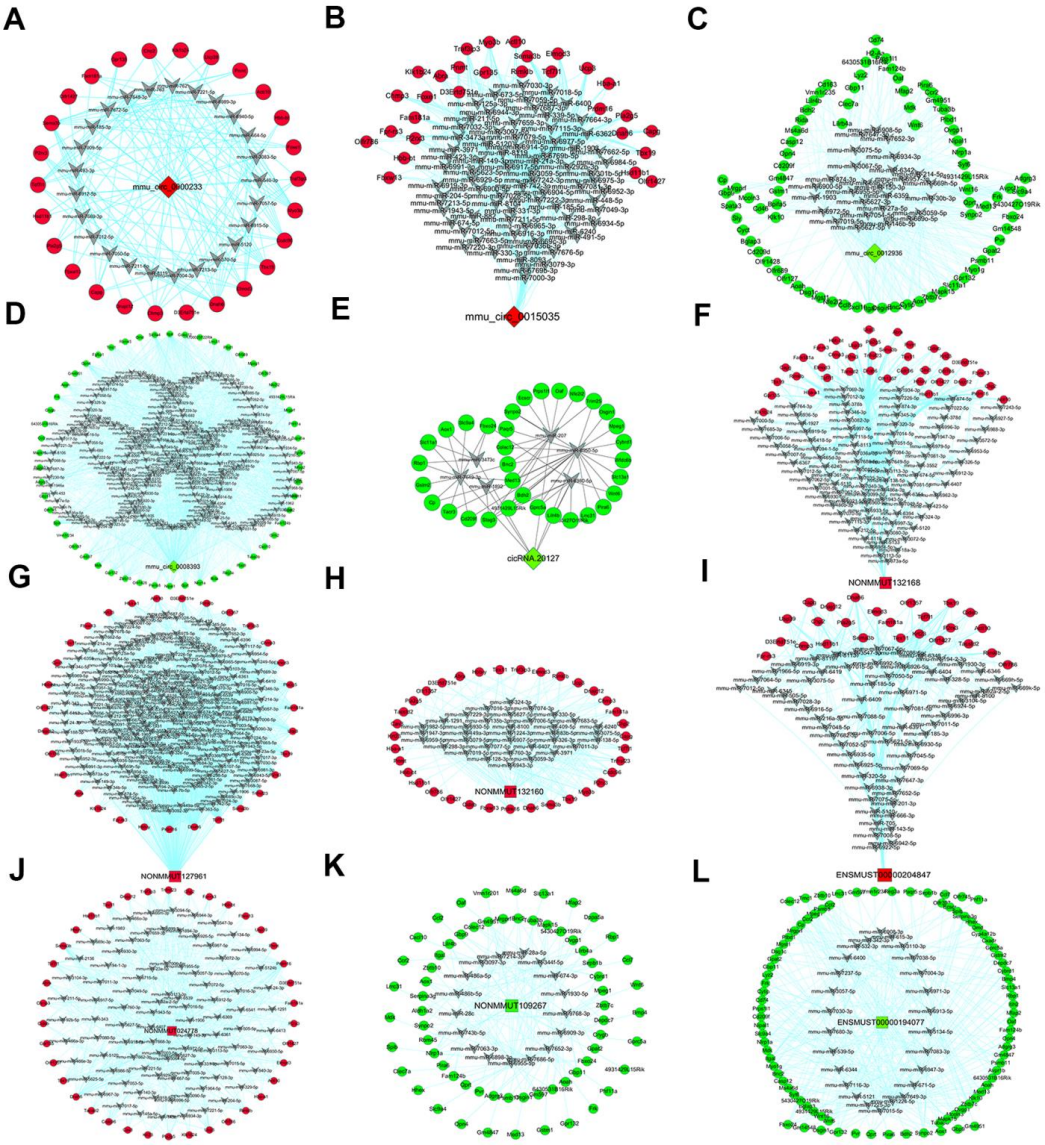
**DEcircRNA/DElncRNA-miRNA-DEmRNA crosstalk network.** Through linear regression model analysis and seed sequence matching method based on gene expression, five DEcircRNAs and seven DElncRNAs were established as a regulatory network of miRNA sponge adsorbents. (**A**–**E**) The network of five verified circRNAs (mmu_circ_0000233 (**A**), mmu_circ_0015035 (**B**), mmu_circ_0012936 (**C**), mmu_circ_0008393 (**D**) and cicRNA.20127 (**E**)). (**F**–**L**) The network of seven verified lncRNAs (NONMMUT132168 (**F**), NONMMUT127961 (**G**), NONMMUT132160 (**H**), ENSMUST00000204847 (**I**), NONMMUT024778 (**J**), NONMMUT109267 (**K**) and ENSMUST00000194077 (**L**)). Red rhombi: up-regulated DEcircRNAs. Green rhombi: down-regulated DEcircRNAs. Red squares: up-regulated DElncRNAs. Green squares: down-regulated DElncRNAs. Gray V: miRNAs. Red circles: up-regulated DEmRNAs. Green circles: down-regulated DEmRNAs.

**Figure 4:**
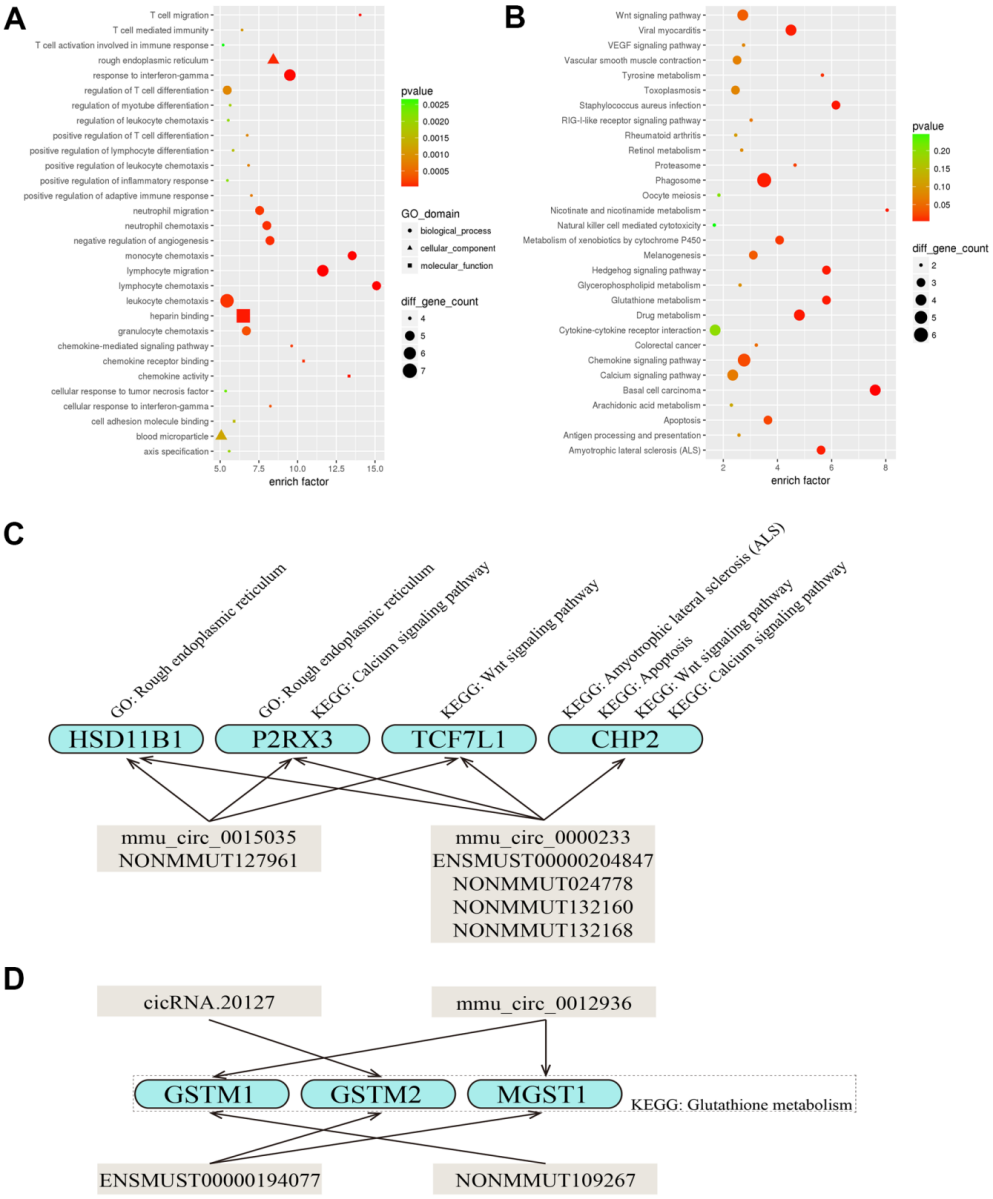
**Predicted functions of the DEcircRNAs/DElncRNAs involved in the DEcircRNA/DElncRNA-miRNA-DEmRNA network.** (**A**, **B**) The top 30 most significantly enriched GO (**A**) and KEGG (**B**) pathways of the DEmRNAs that were targets of the five DEcircRNAs (mmu_circ_0000233, mmu_circ_0015035, mmu_circ_0012936, mmu_circ_0008393 and cicRNA.20127) and seven DElncRNAs (NONMMUT132168, NONMMUT127961, NONMMUT132160, ENSMUST00000204847, NONMMUT024778, NONMMUT109267 and ENSMUST00000194077). The X-axis displays the enrich factor in the GO and KEGG pathways and the Y-axis displays the GO and KEGG pathways. The color scale depicts the *p*-values. The sizes of the nodes represent the counts of genes enriched in the GO and KEGG pathways. (**C**, **D**) Diagram of the various functions of the DEcircRNAs/DElncRNAs.

Twelve of the ncRNAs validated by qRT-PCR (all but mmu_circ_0003404 and NR_045161) were found to be ceRNAs for miRNA targets. When we constructed DEcircRNA/DElncRNA-miRNA-DEmRNA crosstalk networks (Figure 3), we noted that mmu_circ_0000233, ENSMUST00000204847, NONMMUT024778, NONMMUT132160 and NONMMUT132168 shared miRNA response elements (MREs) with four up-regulated DEmRNAs (HSD11B1, P2RX3, TCF7L1 and CHP2) (Figures 3, 4C); mmu_circ_0015035 and NONMMUT127961 also shared MREs with HSD11B1, P2RX3 and TCF7L1 (Figures 3, 4C); and cicRNA.20127, mmu_circ_0012936, ENSMUST00000194077 and NONMMUT109267 shared miRNAs with three down-regulated DEmRNAs (GSTM1, GSTM2 and MGST1) (Figures 3, 4D).

### Functional annotation of DEmRNAs that were targets of DEcircRNAs and DElncRNAs

GO and KEGG pathway analyses were employed to annotate the functions of the DEmRNAs in the DEcircRNA/DElncRNA-miRNA-DEmRNA network. In the GO analysis, the Rough endoplasmic reticulum (GO: 0005791, *p* = 9.090e-05) and Lymphocyte chemotaxis (GO: 0048247, *p* = 4.203e-06) were the most significant cellular component and biological process, respectively (Figure 4A). According to the KEGG pathway analysis, the top 30 most significantly enriched pathways for the DEmRNAs included Glutathione metabolism (KEGG: mmu00480, *p* = 4.435e-03), Amyotrophic lateral sclerosis (ALS) (KEGG: mmu05014, *p* = 5.047e-03), Apoptosis (KEGG: mmu04210, *p* = 2.211e-02), Wnt signaling pathway (KEGG: mmu04310, *p* = 3.932e-02) and Calcium signaling pathway (KEGG: mmu04020, *p* = 6.673e-02) (Figure 4B).

We found that some DEmRNAs (HSD11B1, P2RX3, TCF7L1 and CHP2) that shared MREs with mmu_circ_0000233, mmu_circ_0015035, ENSMUST00000204847, NONMMUT024778, NONMMUT132160, NONMMUT132168 or NONMMUT127961 were enriched in the Rough endoplasmic reticulum (GO: 0005791), Amyotrophic lateral sclerosis (ALS) (KEGG: mmu05014), Apoptosis (KEGG: mmu04210), Wnt signaling pathway (KEGG: mmu04310) or Calcium signaling pathway (KEGG: mmu04020). These findings indicated that circRNAs and lncRNAs both have wide-ranging physiological functions and may co-regulate certain target genes (Figure 4C). We also noted that GSTM1 (sharing miRNAs with mmu_circ_0012936 and NONMMUT109267), GSTM2 (sharing miRNAs with cicRNA.20127 and ENSMUST00000194077) and MGST1 (sharing miRNAs with mmu_circ_0012936 and ENSMUST00000194077) were enriched in the pathway of Glutathione metabolism (KEGG: mmu00480) (Figure 4D).

### Coding potential of DEcircRNAs and DElncRNAs

Among the 197 DEcircRNAs and 685 DElncRNAs, we found that 44 DEcircRNAs and 7 DElncRNAs had scores > 0 in the Coding-Non-Coding Index [27], suggesting that they may have coding potential (Supplementary Table 1).

### Expression correlation analysis between DEcircRNAs/DElncRNAs and their host mRNAs

**Figure 5:**
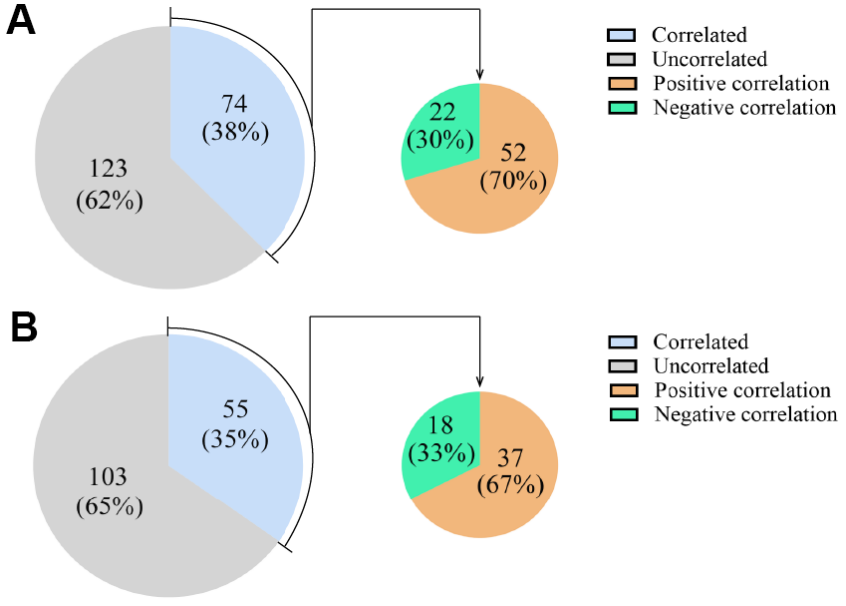
**Expression correlations between DEcircRNAs/DElncRNAs and their host mRNAs.** (**A**) The expression correlations between DEcircRNAs and their host mRNAs. (**B**) The expression correlations between DElncRNAs and their host mRNAs. The blue area represents the DEcircRNAs/DElncRNAs that correlated with their host mRNAs as a percentage of all the DEcircRNAs/DElncRNAs. The gray area represents the remaining DEcircRNAs/DElncRNAs. The orange area represents the DEcircRNAs/DElncRNAs that correlated positively with their host mRNAs, and the green area represents the DEcircRNAs/DElncRNAs that correlated negatively with their host mRNAs, as percentages of the DEcircRNAs/DElncRNAs that correlated with their host mRNAs.

We next evaluated whether the levels of the DEcircRNAs/DElncRNAs correlated with those of their host mRNAs, in order to determine whether the DEcircRNAs/DElncRNAs altered the expression of their host genes. The levels of 38% of the DEcircRNAs (74 of 197) correlated with the levels of their host mRNAs, with 70% correlating positively and 30% correlating negatively (Figure 5A and Supplementary Table 2). After we removed 527 intergenic DElncRNAs without maternal genes, we found that the levels of 35% of the DElncRNAs (55 of 158) correlated with the levels of their host mRNAs, with 67% correlating positively and 33% correlating negatively (Figure 5B and Supplementary Table 2).

### Comparison of DEcircRNAs, DElncRNAs and DEmRNAs among the prefrontal cortex, substantia nigra, corpus striatum and hippocampus

**Figure 6:**
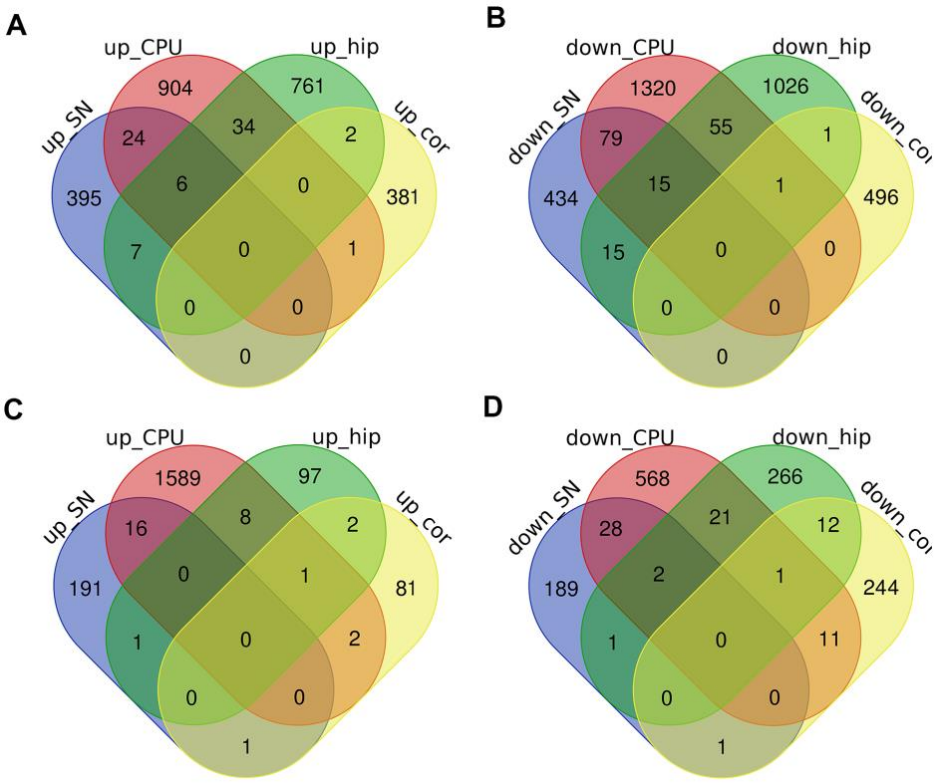
**The intersections of DEcircRNAs, DElncRNAs and DEmRNAs in the prefrontal cortex (cor), substantia nigra (SN), corpus striatum (CPU) and hippocampus (hip).** (**A**–**D**) Venn diagrams of up-regulated DEcircRNAs/DElncRNAs (**A**), down-regulated DEcircRNAs/DElncRNAs (**B**), up-regulated DEmRNAs (**C**) and down-regulated DEmRNAs (**D**) were showed respectively.

Next, we determined the shared DEcircRNAs, DElncRNAs and DEmRNAs among the prefrontal cortex, substantia nigra, corpus striatum and hippocampus, in order to identify common regulatory mechanisms of Nrf2 in these areas. Surprisingly, few circRNAs/lncRNAs were consistently regulated by Nrf2 in all four brain regions. Based on their differential expression between Nrf2 (-/-) and Nrf2 (+/+) mice, no circRNAs/lncRNAs were consistently regulated by Nrf2 in the prefrontal cortex and substantia nigra; only one down-regulated ncRNA (mmu_circ_0010156) was consistently regulated by Nrf2 in the prefrontal cortex, corpus striatum and hippocampus; only one up-regulated ncRNA (ENSMUST00000144818) was consistently regulated by Nrf2 in the prefrontal cortex and corpus striatum; and only two up-regulated ncRNAs (cicRNA.4014 and NR_027831) and one down-regulated ncRNA (ENSMUST00000145790) were consistently regulated by Nrf2 in the prefrontal cortex and hippocampus (Figure 6A, 6B).

While it was surprising that there were so few intersecting DEcircRNAs/DElncRNAs in different brain areas, it was even more interesting that there were few intersecting DEmRNAs. Based on their differential expression between Nrf2 (-/-) and Nrf2 (+/+) mice, only one up-regulated mRNA (THNSL2) and one down-regulated mRNA (MYCT1) were consistently regulated by Nrf2 in the prefrontal cortex and substantia nigra; only one up-regulated mRNA (RBM45) and one down-regulated mRNA (NFE2L2) were consistently regulated by Nrf2 in the prefrontal cortex, corpus striatum and hippocampus; only two up-regulated mRNAs (PRSS56 and SLC29A4), and eleven down-regulated mRNAs (GBP11, FAS, GSTM3, LYZ1, CYBB, CHRNB1, GM14548, GSTM2, C3, PLAC8, and DSG1C) were consistently regulated by Nrf2 in the prefrontal cortex and corpus striatum; and only two up-regulated mRNAs (TEX11 and FAM181A), and twelve down-regulated mRNAs (NAPSA, CYCT, AOX1, SFMBT2, OMD, MRC1, FOXC2, MS4A6B, RIDA, CD163, GSTM1, and MS4A6D) were consistently regulated by Nrf2 in the prefrontal cortex and hippocampus (Figure 6C, 6D). These results indicated that Nrf2 may not have the same function in different brain areas.

## DISCUSSION

The prefrontal cortex is responsible for cognition, emotion, pain and behavior management, and its function is disrupted in various neurodegenerative diseases [28, 29]. There is abundant evidence that insufficient Nrf2 signaling is a major cause of nervous system disorders, particularly neurodegenerative diseases [8, 30–32]. We hypothesized that Nrf2 could exert its neuroprotective effects by altering the expression of circRNAs and lncRNAs. Although circRNAs and lncRNAs differ significantly in shape and size, both are expressed in tissue- and spatiotemporal-specific patterns and regulate their target genes by similar mechanisms [33–36]. Since the ceRNA hypothesis was proposed [37], both circRNAs and lncRNAs have been reported to function as miRNA sponges that alter the stability or translation of mRNAs [21, 38]. Thus, we used microarray and bioinformatics analyses to detect DEcircRNAs and DElncRNAs between the prefrontal cortex tissues of Nrf2 (-/-) and Nrf2 (+/+) mice.

In this study, five DEcircRNAs (cicRNA.20127, mmu_circ_0000233, mmu_circ_0008393, mmu_circ_0012936 and mmu_circ_0015035) and seven DElncRNAs (ENSMUST00000194077, ENSMUST00000204847, NONMMUT024778, NONMMUT109267, NONMMUT127961, NONMMUT132160 and NONMMUT132168) were verified by qRT-PCR and used to construct DEcircRNA/DElncRNA-miRNA-DEmRNA crosstalk networks. Then GO and KEGG pathway analyses were employed to predict their functions. Five of these ncRNAs (mmu_circ_0000233, ENSMUST00000204847, NONMMUT024778, NONMMUT132160 and NONMMUT132168) shared MREs with four up-regulated DEmRNAs (HSD11B1, P2RX3, TCF7L1 and CHP2). HSD11B1 was enriched in the Rough endoplasmic reticulum (GO: 0005791, an organelle that synthesizes new proteins); P2RX3 was enriched in the Rough endoplasmic reticulum (GO: 0005791) and the Calcium signaling pathway (KEGG: mmu04020, an important ion pathway involved in neuronal synaptic signal transmission); TCF7L1 was enriched in the Wnt signaling pathway (KEGG: mmu04310, a key pathway for cell development); and CHP2 was enriched in Amyotrophic lateral sclerosis (ALS) (KEGG: mmu05014, a neurodegenerative disease), Apoptosis (KEGG: mmu04210), Wnt signaling pathway (KEGG: mmu04310) and Calcium signaling pathway (KEGG: mmu04020). Thus, the five aforementioned ncRNAs may contribute to the effects of Nrf2 on cognitive function by regulating protein synthesis, cell degeneration, neuronal development and intersynaptic signal transduction. We also found that mmu_circ_0015035 and NONMMUT127961 could be ceRNAs for HSD11B1, P2RX3 and TCF7L1, suggesting that mmu_circ_0015035 and NONMMUT127961 may assist the above five ncRNAs in regulating of cognitive function in the prefrontal cortex.

In addition, four ncRNAs (cicRNA.20127, mmu_circ_0012936, ENSMUST00000194077 and NONMMUT109267) were found to be ceRNAs for GSTM1, GSTM2 and MGST1. These three DEmRNAs were significantly down-regulated in the prefrontal cortex in Nrf2 (-/-) mice and were enriched in the pathway of Glutathione metabolism (KEGG: mmu00480). GSTM1 and GSTM2 encode glutathione S-transferases, which mainly participate in the phase II detoxification of endogenous oxidative stress products and exogenous electrophilic chemical compounds [39]. Reduced GSTs activity has been observed in multiple brain regions and ventricular cerebrospinal fluid from Alzheimer’s disease patients shortly after death [40]. Furthermore, the cytoprotective effects of Nrf2 are thought to be due to its strong induction of enzymes involved in glutathione synthesis and recycling [41]. MGST1 is a member of the Membrane-Associated Proteins in Eicosanoid and Glutathione metabolism family, an ancient and diverse family thought to predate the cytosolic family of GSTs [42]. MGST1 detoxifies reactive intermediates such as metabolic electrophile intermediates and lipophilic hydroperoxides through its glutathione-dependent transferase and peroxidase activities [43]. Thus, cicRNA.20127, mmu_circ_0012936, ENSMUST00000194077 and NONMMUT109267 may facilitate the effects of Nrf2 on cognitive function by regulating glutathione metabolism.

Interestingly, the results of the GO analysis included immune-related function such as Lymphocyte chemotaxis (GO: 0048247). The central nervous system lacks a classical lymphatic drainage system; however, the discovery of the lymphatic system in the central nervous system in 2015 suggested that central nervous system diseases may be associated with immune system disorders [44–46]. Our results support the notion that the lymphatic system is present in the brain, and indicate that its activity may be influenced by the Nrf2 pathway. However, the mechanisms whereby immune cells enter and exit the central nervous system remain poorly understood.

Although circRNAs and lncRNAs commonly alter gene expression as ceRNAs, when they are present at low levels they may not impact their target miRNAs [47]. CircRNAs and lncRNAs may function through other mechanisms, such as binding with proteins [48, 49] or being translated into proteins/peptides [50, 51]. Interestingly, we identified 44 DEcircRNAs and 7 DElncRNAs with coding potential, in keeping with the view that some ncRNAs may also be mRNAs.

In addition, some reports have indicated that circRNAs and lncRNAs can alter the expression of their host mRNAs [33]. Cis-acting circRNAs and lncRNAs regulate gene expression in a manner dependent on the location of their own transcription sites, at varying distances from their targets in the linear genome [52, 53]. Thus, we analyzed the expression correlations between DEcircRNAs/DElncRNAs and their corresponding host mRNAs. Our results suggested that only a small percentage of DEcircRNAs/DElncRNAs (38% of DEcircRNAs and 35% of DElncRNAs) altered the expression of their host mRNAs. These results were consistent with previous reports indicating that the expression of many circRNAs and lncRNAs is independent of the expression of their related mRNA isoforms [54, 55]. However, other studies have demonstrated that circRNAs are typically generated at the cost of their canonical mRNA isoforms [56, 57].

Among the DEcircRNAs that were correlated with their host mRNAs, only 30% exhibited negative correlations, suggesting that many DEcircRNAs activate transcription. CircRNA, especially exon-intron circRNAs, have been reported to bind specific ally with U1 small nuclear ribonucleoprotein RNA and promote the transcription of their parental genes in cis, although they may also influence other loci in trans [58, 59]. Likewise, the non-random positioning of lncRNAs implies that they are functionally associated with their nearby genes [60]. Cis-acting lncRNAs have been demonstrated to activate, repress or otherwise alter the expression of their target genes through various mechanisms [52]. In the present study, among the DElncRNAs that were associated with their host mRNAs, 67% exhibited positive correlations and 33% negative correlations.

We also determined the shared DEcircRNAs, DElncRNAs and DEmRNAs in four brain regions: prefrontal cortex, substantia nigra, corpus striatum and hippocampus. Interestingly, few circRNAs/lncRNAs were consistently regulated by Nrf2 in these four brain regions, indicating that Nrf2 may not have the same function in different brain areas. Recent studies have demonstrated that alters the expression of not only antioxidant genes, but also genes involved in autophagy, intermediate metabolism, stem cell quiescence and unfolded protein reactions [9]. Therefore, the function of Nrf2 is complex. Moreover, our research suggested that Nrf2 activity may be tissue-specific, which has not been reported before and is worth studying in the future.

In conclusion, our study demonstrated that four DEcircRNAs (mmu_circ_0000233, mmu_circ_0015035, cicRNA.20127 and mmu_circ_0012936) and seven DElncRNAs (ENSMUST00000204847, NONMMUT024778, NONMMUT132160, NONMMUT132168, NONMMUT127961, ENSMUST00000194077 and NONMMUT109267) may contribute to the effects of Nrf2 on cognitive function by regulating protein synthesis, cell degeneration, neuronal development, intersynaptic signal transduction and glutathione metabolism. Our study has laid the foundation for future investigations into the molecular pathways through which Nrf2 protects neurons against oxidative stress and exerts other functions.

## MATERIALS AND METHODS

### Nrf2-knockout mice and ethics

Adult male Nrf2 (+/+) and Nrf2 (-/-) mice (25-30 g, 3-4 months, n = 3) on an ICR background were kindly provided by Professor Chun-Yan Li (Department of Neurology, Second Hospital of Hebei Medical University, Shijiazhuang, China) for this study. PCR amplification of genomic DNA from tails was used to determine the genotypes of the mice. All the mice were sacrificed using an overdose of an isoflurane/oxygen mixture (Huazhong Haiwei Gene Technology Co, Ltd, Beijing, China, 021400). Prefrontal cortex tissues were surgically obtained from each mouse, and immediately homogenized for the extraction of total RNA (TRIzol, Invitrogen, Carlsbad, CA, USA, 15596026).

The animal experiments in this study complied with the regulations of the Animal Welfare Act and the National Institutes of Health Guide for the Care and Use of Laboratory Animals (NIH Publication No. 85-23, revised 1996) and were approved by the ethics committee of Hebei Medical University (IACUC-Hebmu-Glp-2016017).

### Microarray analysis

Six mice (Nrf2 (+/+) group, n = 3; Nrf2 (-/-) group, n = 3) were anesthetized, and their prefrontal cortex tissues were collected and rapidly stored in liquid nitrogen. Total RNA was extracted and purified using an RNeasy Mini Kit (Cat. #74106, QIAGEN, GmbH, Germany) in accordance with the manufacturer’s instructions. Shanghai Biotechnology Corporation (Shanghai, China) performed the quality control for the gene analyses by evaluating the total RNA with a NanoDrop ND-2000 spectrophotometer and an Agilent Bioanalyzer 2100 (Agilent Technologies, Inc., Santa Clara, CA, USA). Only RNA samples that met the quality control standards (RNA integrity number ≥ 7.0 and 28S/18S ≥ 0.7) were included in the subsequent microarray experiments. The included samples were amplified and labeled with a Low Input Quick Amp Labeling Kit, One-Color (Cat. #5190-2305, Agilent Technologies) in accordance with the manufacturer’s instructions. The labeled complementary RNA was purified with an RNeasy Mini Kit (Cat. #74106, QIAGEN).

An SBC Mouse (4*180 K) ceRNA microarray (Shanghai Biotechnology Corporation) was used to detect three types of RNA (circRNA, lncRNA and mRNA). Each slide was hybridized with 1.65 μg of Cy3-labeled complementary RNA using a Gene Expression Hybridization Kit (Cat. #5188-5242, Agilent Technologies) in a Hybridization Oven (Cat. #G2545A, Agilent Technologies) according to the manufacturer’s instructions. After 17 hours of hybridization, the slides were washed in staining dishes (Cat. #121, Thermo Fisher Scientific, Waltham, MA, USA) with a Gene Expression Wash Buffer Kit (Cat. # 5188-5327, Agilent Technologies) and then scanned on an Agilent Microarray Scanner (Cat. #G2565CA, Agilent Technologies) using the default settings. Data were extracted with Feature Extraction software 10.7 (Agilent Technologies). The raw data were normalized with the Quantile algorithm and LIMMA packages in RStudio (http://www.rstudio.com/). DEcircRNAs, DElncRNAs and DEmRNAs with *p*-values < 0.05 and FCs > 1.5 were selected through scatter plots and volcano diagrams using the ‘pheatmap’ package in R language. The data were also visualized through hierarchical clustering analyses.

### qRT-PCR validation

Total RNA was isolated from Nrf2 (-/-) and Nrf2 (+/+) prefrontal cortex tissues using Trizol reagent (Thermo Fisher Scientific, Wilmington, DE, USA) according to the instruction manual, and the RNA quality was examined using 1% agarose gel electrophoresis. Then, the total RNA (2 μg) was reverse-transcribed to cDNA using an M-MLV First Strand Kit (Thermo Fisher Scientific Inc., 00341186) with random primers. The levels of six DEcircRNAs and eight lncRNAs were assessed via qRT-PCR using SuperReal PreMix Plus (SYBR Green) (TIANGEN, China, FP205) on an Illumina Eco PCR machine (Illumina, San Diego, CA, USA). The primers are displayed in Table 3. The qRT-PCR reaction conditions were as follows: an initial denaturation step of 15 min at 95° C, followed by 40 cycles of 15 s at 95° C, 20 s at 55° C, and 20 s at 72° C, and a final step of 5 min at 72° C. For normalization, β-actin was selected as an internal control. All experiments were performed in triplicate. The comparative cycle threshold (2^-ΔΔCT^) method was used to calculate the relative FCs.

**Table 3:** The primers used in qRT-PCR experiments.

**Transcript ID**	**Primers**
mmu_circ_0000233	Forward: 5′ ATCGCCACGCTACCCTCC 3′
	Reverse: 5′ CCAGCCTTTCCCCATCCA 3′
mmu_circ_0015035	Forward: 5′ TTCAGAAGAACAAAGATTACTAC 3′
	Reverse: 5′ CACTGGAACAGGTGAGATG 3′
mmu_circ_0003404	Forward: 5′ TCAACCCAGACCCCAAGT 3′
	Reverse: 5′ TCCTGAGAGTCCACAGAGAAAT 3′
mmu_circ_0012936	Forward: 5′ ATCGTATGGCTTGCCTTC 3′
	Reverse: 5′ GGGGTTCCCTTCTGTGTC 3′
mmu_circ_0008393	Forward: 5′ CAAAACTAAGTGAAAGGC 3′
	Reverse: 5′ GAAAGTCCAGACCATTG 3′
cicRNA.20127	Forward: 5′ CTCCCGCCTGAACTACC 3′
	Reverse: 5′ CACAAACAGCCAACCATC 3′
NONMMUT132168	Forward: 5' CTCATAGACTAAACTTTCACCTT 3'
	Reverse: 5' CCCCTCAAACTAACCATC 3'
NONMMUT127961	Forward: 5' AGAAGGCACTGGACTGGCTC 3'
	Reverse: 5' GAGTTGGAGTTCCGCTGTGAG 3'
NONMMUT132160	Forward: 5' CACTGTGTGTCGCTTGT 3'
	Reverse: 5' GTGACTACTGATAGAGAGGG 3'
NR_045161	Forward: 5' CACATAGAAGCCACACCCA 3'
	Reverse: 5' AGTCCTCCACCTCGCCT 3'
ENSMUST00000204847	Forward: 5' TGACCACTTGGGGGAAAT 3'
	Reverse: 5' AAGAGGCTGCTTAGAAAAACA 3'
NONMMUT024778	Forward: 5' CCACTGTTTTGTCGGGGT 3'
	Reverse: 5' CTGGGTGTTGGGGCTATT 3'
NONMMUT109267	Forward: 5' TGCGATGTGTTCTGAGTT 3'
	Reverse: 5' TGTGTCCTGCTGTTCTTT 3'
ENSMUST00000194077	Forward: 5' ATCATTTTCTCTTATCAT 3'
	Reverse: 5' TTACTCTCACTTTCTCTC 3'
β-actin	Forward: 5' TCATCACTATTGGCAACGAGCGGT 3'
	Reverse: 5' GTGTTGGCATAGAGGTCTTTACG 3'

qRT-PCR, quantitative real-time polymerase chain reaction.

### Construction of ceRNA networks based on DEcircRNA/DElncRNA-miRNA-DEmRNA interactions

DEcircRNAs and DElncRNAs verified by qRT-PCR were selected for the construction of interaction networks by Shanghai Biotechnology Corporation. First, a DEcircRNA/DElncRNA-DEmRNA co-expression analysis was performed based on the Pearson correlation coefficients between DEmRNA and DEcircRNA/DElncRNA levels in the SBC Mouse (4*180K) ceRNA Array analysis. Genes with Pearson correlation coefficients > 0.81 and *p*-values < 0.05 were recommended for further analysis. Then, DEcircRNA/DElncRNA-miRNA-DEmRNA interaction networks were constructed using miRanda, and binding sites with relatively high scores (≥ 140) were identified. Finally, the networks were visualized using Cytoscape_v3.7.0 (http://www.cytoscape.org/).

### Functional annotation of DEmRNAs that were targets of DEcircRNAs and DElncRNAs

GO and KEGG pathway enrichment analyses were conducted for the target DEmRNAs of miRNAs sponged by the DEcircRNAs/DElncRNAs in the prefrontal cortex tissues. The analyses were performed using the clusterProfiler of R/bioconductor (http://enrich.shbio.com/index/ga.asp) to determine the potential functions of the DEcircRNAs and DElncRNAs.

### Coding potential of DEcircRNAs and DElncRNAs

The Coding-Non-Coding Index (https://github.com/www-bioinfo-org/CNCI) [27], which distinguishes between coding and noncoding transcripts based on their triplet base composition, was used to predict the protein coding potential of the DEcircRNAs and DElncRNAs (The full-length sequences of some of the DEcircRNAs have yet to be confirmed, so the sequences within 300 base pairs around the cut site were used to predict their coding potential).

### Expression correlation analysis between DEcircRNAs/DElncRNAs and their host mRNAs

Pearson correlation coefficients were used to determine the expression correlations between DEcircRNAs/DElncRNAs and mRNAs encoded by the same parental genes. DEcircRNA/DElncRNA-host mRNA pairs with *p*-values < 0.05 were retained.

### Comparison of DEcircRNAs, DElncRNAs and DEmRNAs among the prefrontal cortex, substantia nigra, corpus striatum and hippocampus

DEcircRNAs, DElncRNAs and DEmRNAs in the substantia nigra, corpus striatum and hippocampus were screened in our previous studies of Nrf2 (-/-) and Nrf2 (+/+) mice [23–25]. The shared DEcircRNAs, DElncRNAs and DEmRNAs among these areas and the prefrontal cortex were determined using the Bioinformatics and Evolutionary Genomics tool (http://bioinformatics.psb.ugent.be/webtools/Venn/).

### Statistical analysis

All data were analyzed using SPSS 22.0 software (IBM). The means and standard deviations were determined, and independent-samples t-test were performed. *P*-values < 0.05 was considered significant.

## Supplementary Material

Supplementary Figure 1

Supplementary Table 1

Supplementary Table 2
