# Supplementary material for: Functional predication of differentially expressed circRNAs/lncRNAs in the prefrontal cortex of Nrf2-knockout mice
Source: Aging (Albany NY). 2021 Mar 10;13(6):8797–816. doi: 10.18632/aging.202688 (PMC8034947; doi:10.18632/aging.202688)
Supplement: Supplementary Figure 1 [file aging-13-202688-s001.pdf]

SUPPLEMENTARY FIGURE

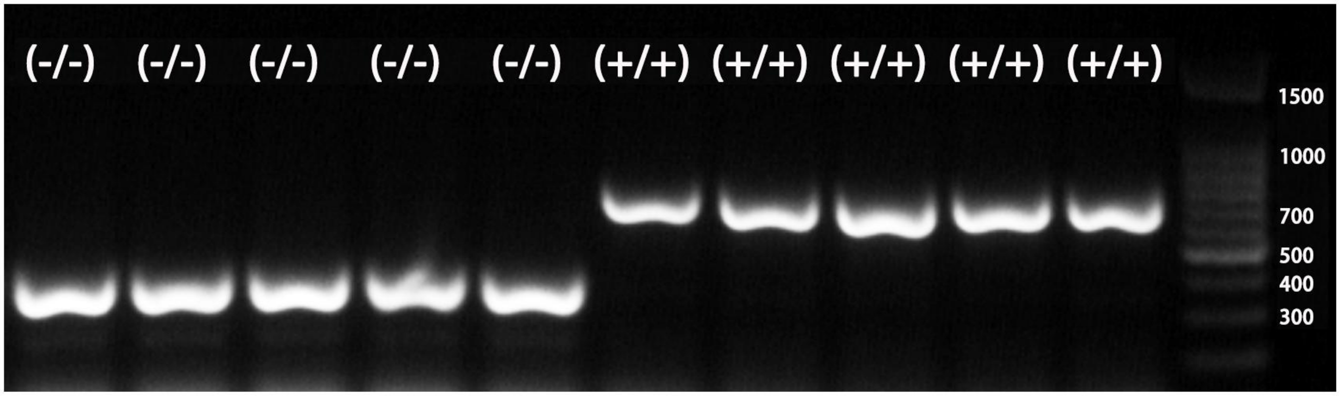

Supplementary Figure 1. Agarose gel electrophoresis for Nrf2 genotype identification. A single band around 400 base pairs identifies Nrf2 (-/-) mice, and a single band around 700 base pairs identifies Nrf2 (+/+) mice.
