# Supplementary material for: Functional predication of differentially expressed circRNAs/lncRNAs in the prefrontal cortex of Nrf2-knockout mice
Source: Aging (Albany NY). 2021 Mar 10;13(6):8797–816. doi: 10.18632/aging.202688 (PMC8034947; doi:10.18632/aging.202688)
Supplement: Supplementary Table 1 [file aging-13-202688-s002.pdf]

SUPPLEMENTARY TABLE

Supplementary Table 1. DEcircRNAs and DElncRNAs with coding potential.

Transcript ID	Type	index	score	start	end	length
cicRNA.8466	circRNA	coding	0.495	0	147	300
cicRNA.22210	circRNA	coding	0.492	0	144	300
mmu_circ_0011393	circRNA	coding	0.462	0	297	452
mmu_circ_0003404	circRNA	coding	0.46	0	198	351
mmu_circ_0000294	circRNA	coding	0.453	0	186	341
cicRNA.9167	circRNA	coding	0.405	0	144	300
cicRNA.26879	circRNA	coding	0.398	0	126	300
mmu_circ_0010317	circRNA	coding	0.391	0	186	5914
cicRNA.21900	circRNA	coding	0.387	0	105	300
mmu_circ_0006643	circRNA	coding	0.365	351	732	887
cicRNA.9806	circRNA	coding	0.351	0	147	300
mmu_circ_0007747	circRNA	coding	0.35	0	153	393
cicRNA.22949	circRNA	coding	0.341	0	147	300
cicRNA.14293	circRNA	coding	0.31	0	147	300
mmu_circ_0000233	circRNA	coding	0.299	0	204	360
mmu_circ_0005741	circRNA	coding	0.284	0	447	602
cicRNA.13720	circRNA	coding	0.283	0	144	300
mmu_circ_0002377	circRNA	coding	0.283	0	996	1150
cicRNA.1182	circRNA	coding	0.281	0	144	300
cicRNA.24048	circRNA	coding	0.261	0	147	300
mmu_circ_0006760	circRNA	coding	0.255	0	327	481
cicRNA.9803	circRNA	coding	0.243	0	147	300
mmu_circ_0003323	circRNA	coding	0.242	147	957	1111
mmu_circ_0007093	circRNA	coding	0.224	0	585	786
mmu_circ_0006074	circRNA	coding	0.221	0	189	344
cicRNA.23286	circRNA	coding	0.21	0	144	300
cicRNA.6161	circRNA	coding	0.203	0	111	300
cicRNA.5874	circRNA	coding	0.202	0	135	300
mmu_circ_0006597	circRNA	coding	0.19	0	3549	3704
cicRNA.8829	circRNA	coding	0.163	0	144	300
mmu_circ_0008393	circRNA	coding	0.16	0	2178	2677
mmu_circ_0010608	circRNA	coding	0.16	0	252	405
mmu_circ_0010196	circRNA	coding	0.146	3057	3747	3901
mmu_circ_0004041	circRNA	coding	0.144	0	1299	1453
mmu_circ_0010157	circRNA	coding	0.142	0	369	523
mmu_circ_0012936	circRNA	coding	0.134	0	783	936
cicRNA.2029	circRNA	coding	0.108	0	144	300
mmu_circ_0010156	circRNA	coding	0.106	189	1284	1438
cicRNA.17816	circRNA	coding	0.09	0	144	300
cicRNA.9831	circRNA	coding	0.058	0	147	300
cicRNA.2059	circRNA	coding	0.05	21	144	300
cicRNA.16687	circRNA	coding	0.026	0	147	300
mmu_circ_0015035	circRNA	coding	0.007	1752	1896	2051
cicRNA.3545	circRNA	coding	0.001	0	129	300
NR_003634	lncRNA	coding	0.599	0	723	941
ENSMUST00000193778	lncRNA	coding	0.269	0	270	2174

ENSMUST00000207903	lncRNA	coding	0.126	0	879	1038
ENSMUST00000077153	lncRNA	coding	0.079	129	1191	1983
ENSMUST00000190980	lncRNA	coding	0.077	0	150	534
NR_015490	lncRNA	coding	0.076	0	246	763
ENSMUST00000212669	lncRNA	coding	0.059	825	1137	2443
